# Identification of IFN-γ-producing T cells as the main mediators of the side effects associated to mouse interleukin-15 sustained exposure

**DOI:** 10.18632/oncotarget.10264

**Published:** 2016-06-23

**Authors:** Marianna Di Scala, Irene Gil-Fariña, Cristina Olagüe, Africa Vales, Luciano Sobrevals, Puri Fortes, David Corbacho, Gloria González-Aseguinolaza

**Affiliations:** ^1^ Gene Therapy and Regulation of Gene Expression Program, Center for Applied Medical Research (CIMA), Pamplona, Spain; ^2^ Imaging Unit and Cancer Imaging Laboratory, Center for Applied Medical Research (CIMA), University of Navarra, Pamplona, Spain; ^3^ Department of Translational Oncology, National Center for Tumor Diseases (NCT) and German Cancer Research Center (DKFZ), Heidelberg, Germany

**Keywords:** interleukin-15, side effects, interferon-γ, T cells, genetic transfer, Immunology and Microbiology Section, Immune response, Immunity

## Abstract

Interleukin-15 (IL-15) is a cell growth-factor that regulates lymphocyte function and homeostasis. Its strong immunostimulatory activity coupled with an apparent lack of toxicity makes IL-15 an exciting candidate for cancer therapy, somehow limited by its short half-life in circulation. To increase IL-15 bioavailability we constructed a recombinant adeno-associated vector expressing murine IL-15 (AAV-mIL15) in the liver. Mice injected with AAV-mIL15 showed sustained and vector dose-dependent levels of IL-15/IL-15Rα complexes in serum, production of IFN-γ and activation of CD8+ T-cells and macrophages. The antitumoral efficacy of AAV-mIL15 was tested in a mouse model of metastatic colorectal cancer established by injection of MC38 cells. AAV-mIL15 treatment slightly inhibits MC38 tumor-growth and significantly increases the survival of mice. However, mIL-15 sustained expression was associated with development of side effects like hepatosplenomegaly, liver damage and the development of haematological stress, which results in the expansion of hematopoietic precursors in the bone marrow. To elucidate the mechanism, we treated IFN-γ receptor-, RAG1-, CD1d- and μMT-deficient mice and performed adoptive transfer of bone marrow cells from WT mice to RAG1-defcient mice. We demonstrated that the side effects of murine IL-15 administration were mainly mediated by IFN-γ-producing T-cells.

**Conclusion:**

IL-15 induces the activation and survival of effector immune cells that are necessary for its antitumoral activity; but, long-term exposure to IL-15 is associated with the development of important side effects mainly mediated by IFN-γ-producing T-cells. Strategies to modulate T-cell activation should be combined with IL-15 administration to reduce secondary adverse events while maintaining its antitumoral effect.

## INTRODUCTION

Interleukin 15 (IL-15) is a γ-chain (γc) cytokine similar to Interleukin 2 (IL-2) [1, 2]. IL-2 is a cytokine with strong antitumoral effect that has been used in the clinic for the treatment of metastatic melanoma and metastatic renal cell carcinoma. However, despite its antitumoral activity in some patients, IL-2 often produces serious side effects that compromise the treatment, or even result in the patient's death. IL-15, has been proposed as a promising candidate to replace IL-2 for cancer therapy [3, 4]. IL-15 is a potent immunostimulatory cytokine. Multiple cell types, like monocytes/macrophage, dendritic cells or endothelial cells produce IL-15 in response to different stimuli [5-7]. IL-15 induces the proliferation, differentiation and activation of CD8+ T-cells [8], and the maintenance of CD8+ memory T-cells. Interestingly, IL-15 inhibits the activation-induced cell death (AICD) of T-cells and is not involved in the maintenance of CD4+CD25+ regulatory T (Treg) cells while IL-2 has been shown to induce AICD and is required for Treg proliferation and survival [9]. IL-15 is an essential factor for the development, survival [10-12] and also to promote increased killer-function of NK and NKT cells activity. Furthermore, IL-15 induces B cells proliferation, antibody production [13] and promotes antibody-dependent cellular cytotoxicity (ADCC) [14, 15]. In addition, IL-15 activates also the secretion of other immunostimulatory cytokines such as IFN-γ and TNF-α [14, 16].

All these functions together with animal testing demonstrating the ability of IL-15 to mediate therapeutic effects in murine transplanted tumors [17, 18] has generated a great deal of interest in bringing this agent to clinical testing in cancer patients [19, 20]. In fact, IL-15 was ranked first among different agents with high potential as antitumoral drug [21] and clinical trials in which this cytokine is given systemically are currently ongoing (NCT01369888, NCT01385423, NCT01021059, NCT01572493, NCT0196789, NCT02099539, NCT01885897). A report containing the data from the first in human clinical trial of recombinant-IL15 (rIL-15) has been recently published, showing a moderate antitumoral efficacy. However, hematological side effects and liver damage, particularly, at the highest dose (3 μg/kg) were reported [22]. In order to avoid rIL15-mediated side effects the investigators proposed alternative dosing strategies such as continuous intravenous infusion.

Drawbacks of recombinant protein include that bolus administration of IL-15 might result in multiple “peaks and valleys” pharmacokinetics. Furthermore, IL-15 as a soluble molecule undergoes rapid renal clearance and has to be administrated daily in clinical trial. This could easily result in both toxicity and suboptimal efficacy. In addition, for effective signaling, IL-15 needs to be bound to IL-15 receptor a subunit (IL-15Ra), a high affinity receptor, which allows IL-15 to interact with the IL-2Rβγ_c_ heterodimeric intermediate affinity receptor present on the responder immune cells [23].

Trying to overcome these limitations we have taken advantage of the use of gene transfer strategies. Among the viral-based vectors employed in gene therapy, recombinant adeno-associated virus (rAAV) is one of the most promising since it has many attractive features including demonstrated safety, efficacy and long term expression in humans [24-26]. rAAV can stably transduced different types of cells and organs. In this work we have developed an AAV serotype 8 vector carrying the native form of interleukin-15 cDNA (AAV-mIL15) under the control of a liver specific promoter [27].

Metastatic liver cancer is a life-threatening condition frequently observed in a significant percentage of colorectal cancer patients. Hepatic lesions are found in 10 to 25% of colorectal cancer patients at the time of diagnoses [28]. In addition, recurrence after surgical removal of the colorectal tumor occurs mainly in the liver, with a 20-25% rate of liver metastases. Surgical resection is potentially curative only in the most favorable cases, whereas chemotherapy and regional treatments achieve local control, but a significant increase in long-term survival is not guaranteed [29]. It is clear that new therapeutic options are needed to improve the clinical management of hepatic metastases from colon cancer.

We have evaluated the antitumoral activity of prolonged hepatic expression of IL-15, using a recombinant AAV vector as a gene delivery vehicle, in a mouse model of liver metastasis derived from colorectal cancer by intrahepatic injection of MC38 cells in C57BL/6 syngeneic mice. We found that in hepatocytes IL-15 is synthesized along with IL15-Rα and its expression results in the activation of T cells and macrophages. Interestingly while we observed a continuous expansion of CD8+ T cells, NK cells after a transient increase, immediately disappear from all the organs analyzed. Treatment with AAV-mIL15 of MC38 tumor-bearing mice results in a T cell independent moderate anti-tumoral effect. However, we also found that long-term exposure to mIL-15 is associated with the development of hepatosplenomegaly and hematological toxicity mainly mediated by IFN-γ producing T cells.

## RESULTS

### AAV-mIL15 administration is associated with the presence of IL-15/IL-15Rα complex in serum and IFN-γ production in a vector dose-dependent manner

**Figure 1:**
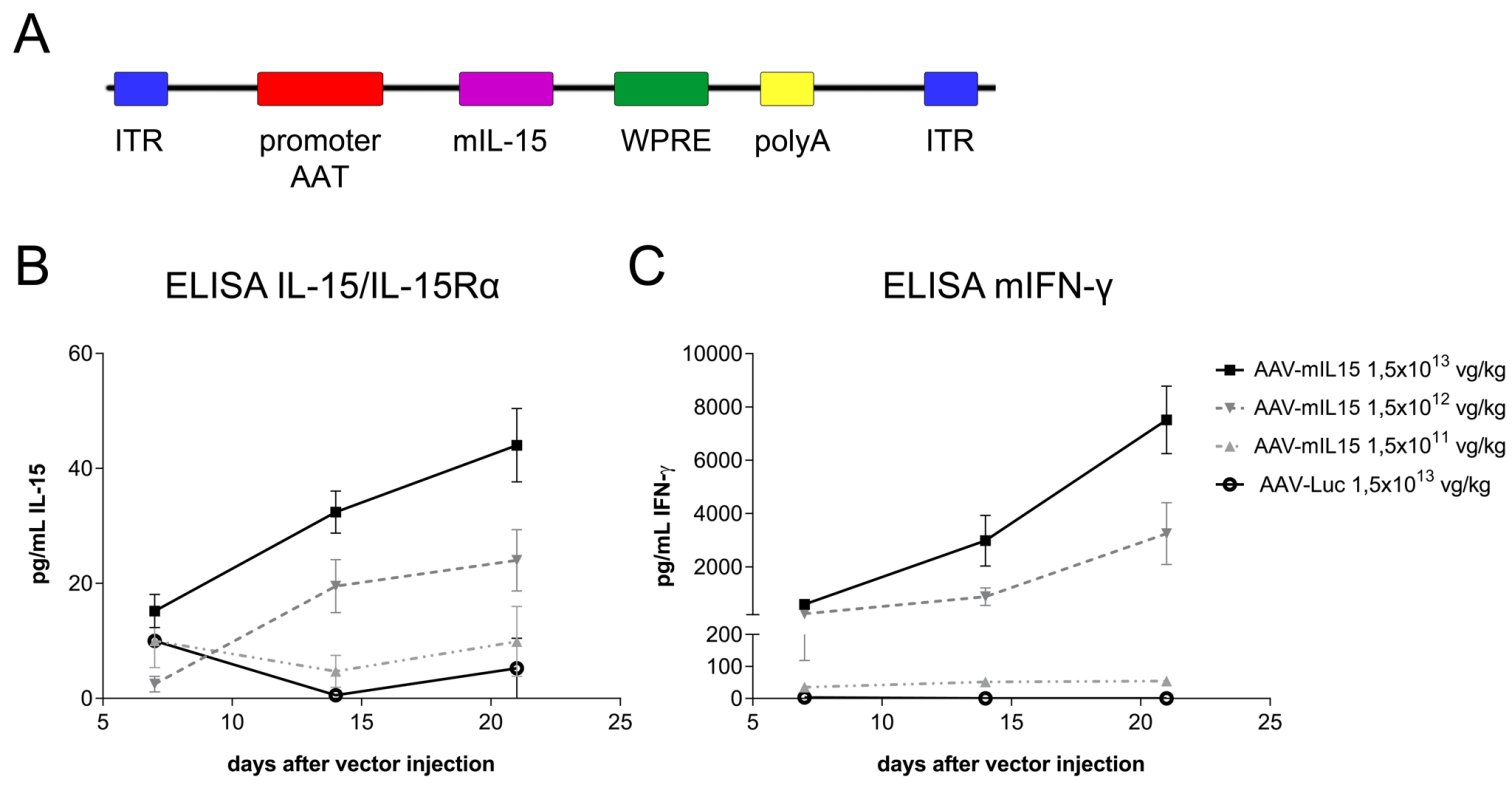
*In vivo* characterization of AAV-mIL15 **A.** Schematic diagram of adeno-associated viral (AAV) vectors used in this study. α1-anti-trypsin (AAT) promoter, Albumin enhancer (Ealb); inverted terminal repeat (ITR); Woodchuck Hepatitis Virus Posttranscriptional Regulatory Element (WPRE); SV40 poly-A fragment containing the early and late polyadenylation signals (pA). For *in vivo* characterization C57BL/6 male mice received 1.5 × 10^13^, 1.5 × 10^12^, 1.5 × 10^11^ vg/kg of AAV-mIL15 or 1.5 × 10^13^ vg/kg of AAV-Luc (*n* = 6-8). IL-15/IL-15Rα complexes **B.** and IFN-γ **C.** concentration was measured in serum by enzyme-linked immunosorbent assay (ELISA) every week for three weeks after AAV administration. Results are expressed as the mean ± SD of 6-8 animals per group.

We constructed and produced a recombinant AAV serotype 8 expressing mIL-15 bearing the native long signal peptide under the regulation of a chimeric liver-specific promoter composed of the human α1-antitrypsin promoter (AAT) with regulatory sequences from the albumin enhancer (Ealb) AAV-mIL15 (Figure 1A) [27]. To test the functionality of the vector C57BL/6 male mice (*n* = 8) were intravenously injected with three different doses of AAV-mIL15: 1.5 × 10^11^, 1.5 × 10^12^, and 1.5 × 10^13^ viral genomes (vg)/kg. A control group was injected with 1.5 × 10^13^ vg/kg of an AAV8 expressing luciferase under the control of the same promoter (AAV-Luc). mIL-15 and IFN-γ expression was analyzed in serum by ELISA, 7, 14, and 21 days after AAV administration. No mIL-15 was detected in serum when the determination was performed using a commercial ELISA recognizing IL-15 (data not shown), however, dose dependent mIL-15 levels were determined using an ELISA that detects the complex IL-15/IL-15Rα, indicating that the recombinant mIL-15 expressed by hepatocytes is present in the blood bound to the IL-15Rα subunit (Figure 1B). As shown in Figure 1C, IFN-γ production correlates with IL-15/IL-15Rα expression levels.

### mIL-15 hepatic expression changes the composition of lymphocyte populations in different organs and tissues

**Figure 2:**
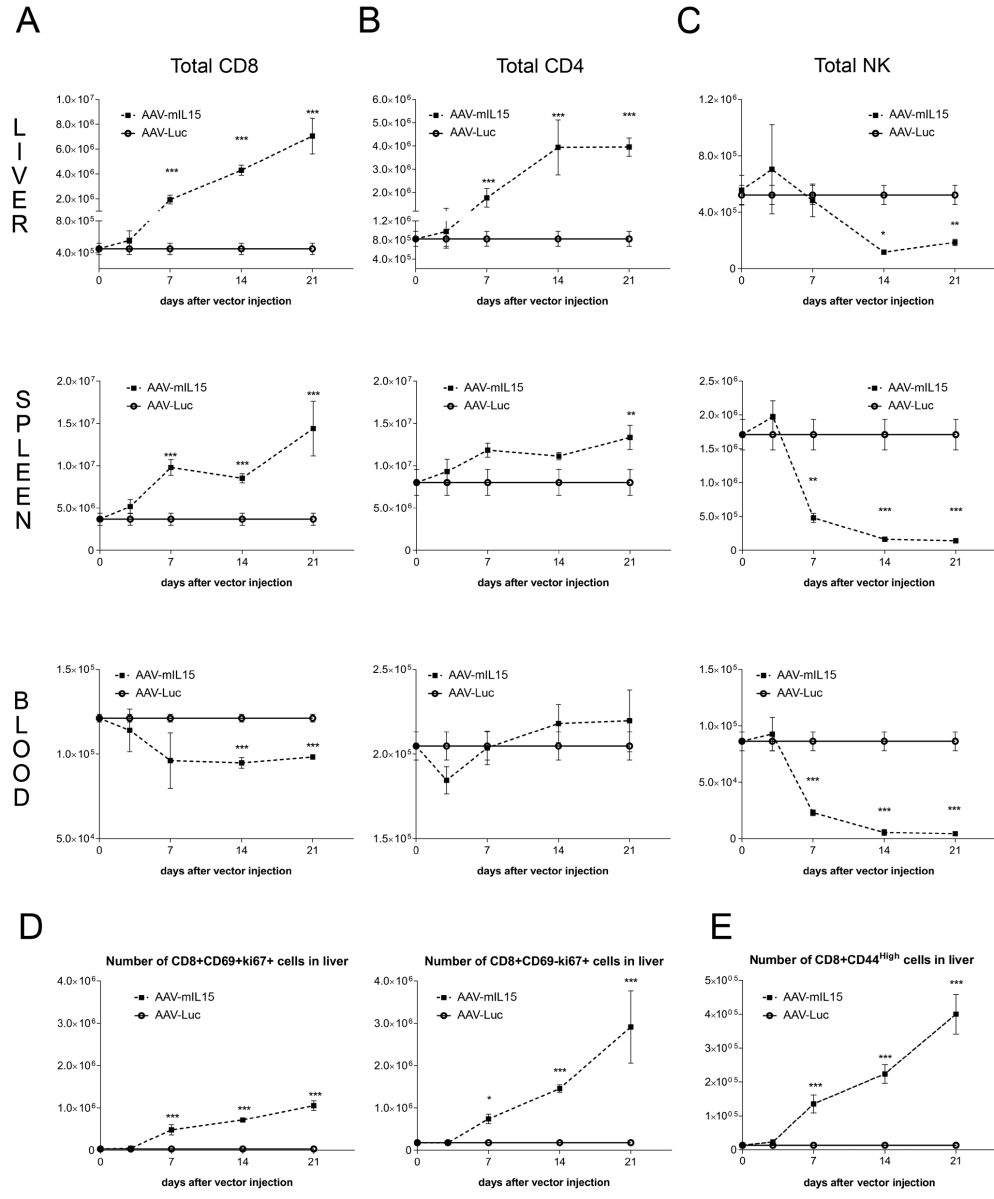
Analysis of lymphocyte subsets in liver, spleen and blood after administration of AAV-mIL15 A-C. 3, 7, 14, and 21 days after AAV-mIL15 or AAV-Luc administration mice were sacrificed and the number of CD8 **A.** CD4 **B.** and NK **C.** cells obtained from the liver, spleen and blood was analyzed by flow cytometry. **D.** At the same time points the numbers of CD8+ CD69+ (resident cells) and CD8+ CD69- proliferating cells (Ki-67+) were determined in the liver. **E.** Activation status of CD8+ was determined by analyzing the expression of the activation marker CD44. For each panels the results are expressed as the mean ± SD of the values obtained from 6-8 mice per group.

Flow cytometry analysis at day 21 of the lymphocyte populations in the liver of animals treated with 1.5 × 10^13^ vg/kg of AAV-mIL15 revealed a significant increase in absolute numbers of CD8+ and CD4+ T cells and a significant decrease of NK1.1+ cells in the liver (Supplementary Figure S1A). AAV-mIL15 treatment inverted the CD4/CD8 T-cell ratio (Supplementary Figure S1B). Since IL-15 induces NK and NKT cell proliferation and survival, the reduction of NK1.1+ cells was surprising. Thus, 3, 7, 14 and 21 days after the administration of AAV-mIL15 or AAV-Luc we analysed the absolute numbers of CD4, CD8 and NK positive cells in the liver, spleen, peripheral blood, bone marrow and lymph nodes. We observed a significant and sustained increase in the absolute numbers of both CD4+ and CD8+ T cells in the liver and in the spleen (Figure 2A and 2B), while NK cells showed a moderate increase at day 3 in both organs abruptly and significantly decreasing thereafter (Figure 2C). In peripheral blood absolute CD8+ T cells numbers decreased immediately after the treatment reaching stable levels at day 7, while CD4+ T cells initially decreased (day 3) and then increased at day 7 reaching normal levels (Figure 2A and 2B). NK cells slightly increased at day 3 but immediately decreased as observed in the liver and in the spleen (Figure 2C). In the bone marrow we observed an increase in CD8+ T cells, a non-significant reduction of CD4+ T cells and a significant reduction of NK1.1+ cells, while in the lymph nodes all three cell types increased at day 3, decreasing thereafter below normal levels (Supplementary Figure S1C). Taking together all these data we can conclude that long-term IL-15 exposure induces a dramatic reduction of NK1.1+ cells in all the compartments analysed.

**Figure 3:**
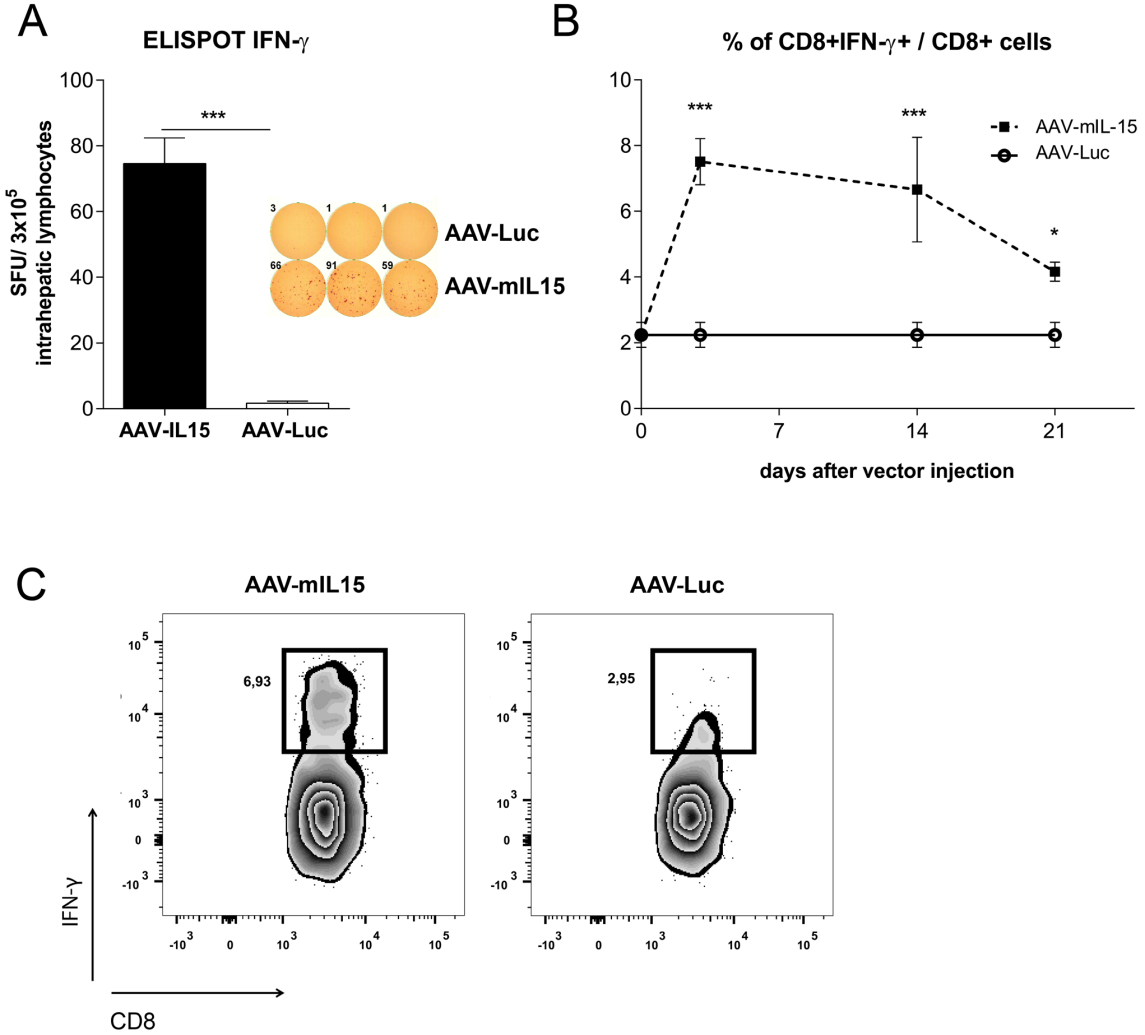
Analysis of IFN-γ production by liver lymphocytes obtained from AAV-mIL5 treated mice **A.** 21 days after AAV-mIL15 or AAV-Luc administration, mice were sacrificed and liver lymphocytes were isolated. The number of cells producing IFN-γ was determined using an Elispot assay. **B.** 3, 14 and 21 liver lymphocytes were isolated from AAV-mIL15 and AAV-Luc treated mice and IFN-γ production by CD8+ T cells was analysed after PMA + Ionomycin stimulation by intracellular staining and flow cytometry analysis. **C.** Representative images of flow cytometry analysis of CD8+ T cells producing IFN-γ.

Since CD8+ T cells significantly increased in the liver we analysed the proliferation of CD8+ CD69+ (considered as resident CD8 cells) and CD8+CD69- cells by Ki67 staining. As shown in Figure 2D, we observed a high proliferation in both subsets. Furthermore, a significant increase in activated CD8+ T cells (CD8+ CD44^hi^) was observed in the liver of AAV-mIL15 treated mice (Figure 2E and Supplementary Figure S1D). Moreover, we analysed IFN-γ production by liver intrahepatic lymphocytes obtained from mice 21 days after the administration of 1.5 × 10^13^ vg/kg of AAV-mIL15 or AAV-Luc in an Elispot assay and found that the number of IFN-γ producing cells was significantly higher in mice receiving mIL15 than in AAV-Luc treated mice (Figure 3A). Additionally, we analysed IFN-γ production by CD8+ T cells obtained from the liver 3, 14 and 21 days after the administration of AAV-mIL15 or AAV-Luc by intracellular staining followed by flow cytometry analysis and as shown in Figure 3B, 3C we observed a significant increase of IFNγ-producing CD8+ T cells in the liver of mice treated with IL-15.

### AAV-mediated mIL-15 expression in MC38 tumor results in a prolonged survival with no reduction in tumor burden

After testing the capacity of AAV-mIL15 to induce CD8 T cell expansion and activation we tested the antitumoral activity of this vector in MC38 tumor-bearing mice. MC38 cells were subcutaneously or intrahepatically injected in C57BL/6 mice (*n*8-12) and in both cases AAV-mIL15 or AAV-Luc was administered intravenously at a dose of 1.5×10^13^ vg/kg one week after tumor cell injection. The size of subcutaneously implanted MC38 tumors was measured every three days after vector injection, and the size of intrahepatic tumors was measured 10 days after vector injection by ecography. Survival was checked daily and mice were euthanized if general status was deteriorated or subcutaneous tumors exceeded 20 mm in diameter.

**Figure 4:**
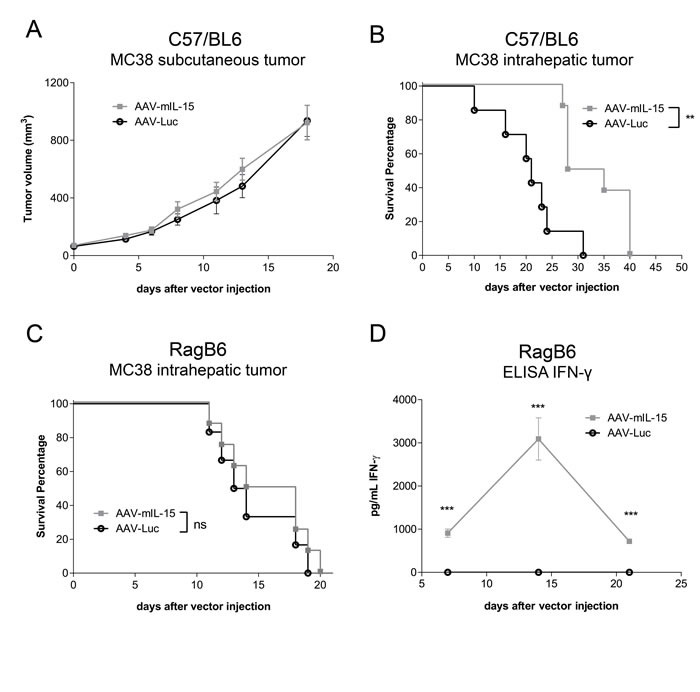
Analysis of the antitumoral effects of AAV-mIL15 on MC38 tumor **A.** Tumor volume follow-up of the subcutaneous MC38 tumor model. Results are expressed as the mean ± SD. 10-12 mice per group. **B.** Kaplan-Meier survival analysis of C57BL/6 WT mice with established MC38 tumors after treatment is assessed (**p <0.01). 8 mice per group. **C.** Kaplan-Meier survival analysis of RAG1−/− mice with established MC38 tumors after treatment is assessed (ns). **D.** IFN-γ concentration was measured in serum by enzyme-linked immunosorbent assay (ELISA) in RAG1−/− mice every week for three weeks after AAV administration. Results are expressed as the mean ± SD of of the values obtained from 6-8 mice per group.

In the case of subcutaneous MC38 tumor injection, no difference in tumor growth was observed between the groups receiving AAV-mIL15 or AAV-Luc, indicating that mIL-15 produced in the liver failed to inhibit or delay tumor growth (Figure 4A). However, when the tumor was implanted intrahepatically, a slight reduction in tumor growth was observed at day 10 after vector injection in the animals receiving AAV-mIL15 in comparison to AAV-Luc treated mice (AAV-Luc-group: 116.1 ± 41.02 mm^3^ vs AAV-mIL-15-group: 77.08 ± 42.36 mm^3^). Furthermore, thirty days after cell implantation all the animals from the control group were dead as a consequence of tumor progression, in contrast, 50% of the mice receiving AAV-mIL15 were alive (Figure 4B). At day 40 after AAV injection all mice receiving mIL-15 had to be sacrificed due to clear signs of ill health. To determine the role of T cells in the antitumoral effect of AAV-mIL15 we repeated the experiment implanting MC38 tumors in RAG1−/− mice and tumor growth and survival was evaluated as previously indicated. Although a reduction in tumor growth was observed at day 7 after vector injection in animals having received AAV-mIL15 (data not shown), no differences in survival was observed (Figure 4C), indicating that the initial antitumoral effect is T cell independent while the maintenance of the effect requires the presence of T cells. Interestingly, contrary to the sustained IFN-γ expression observed in C57BL/6 mice, in RAG1−/− mice it reached a peak at day 14 and declined thereafter (Figure 4D), indicating that sustained IFN-γ expression depends on the presence of T cells.

### Sustained IL-15/IL-15Rα expression induces hepato- and splenomegaly

**Figure 5:**
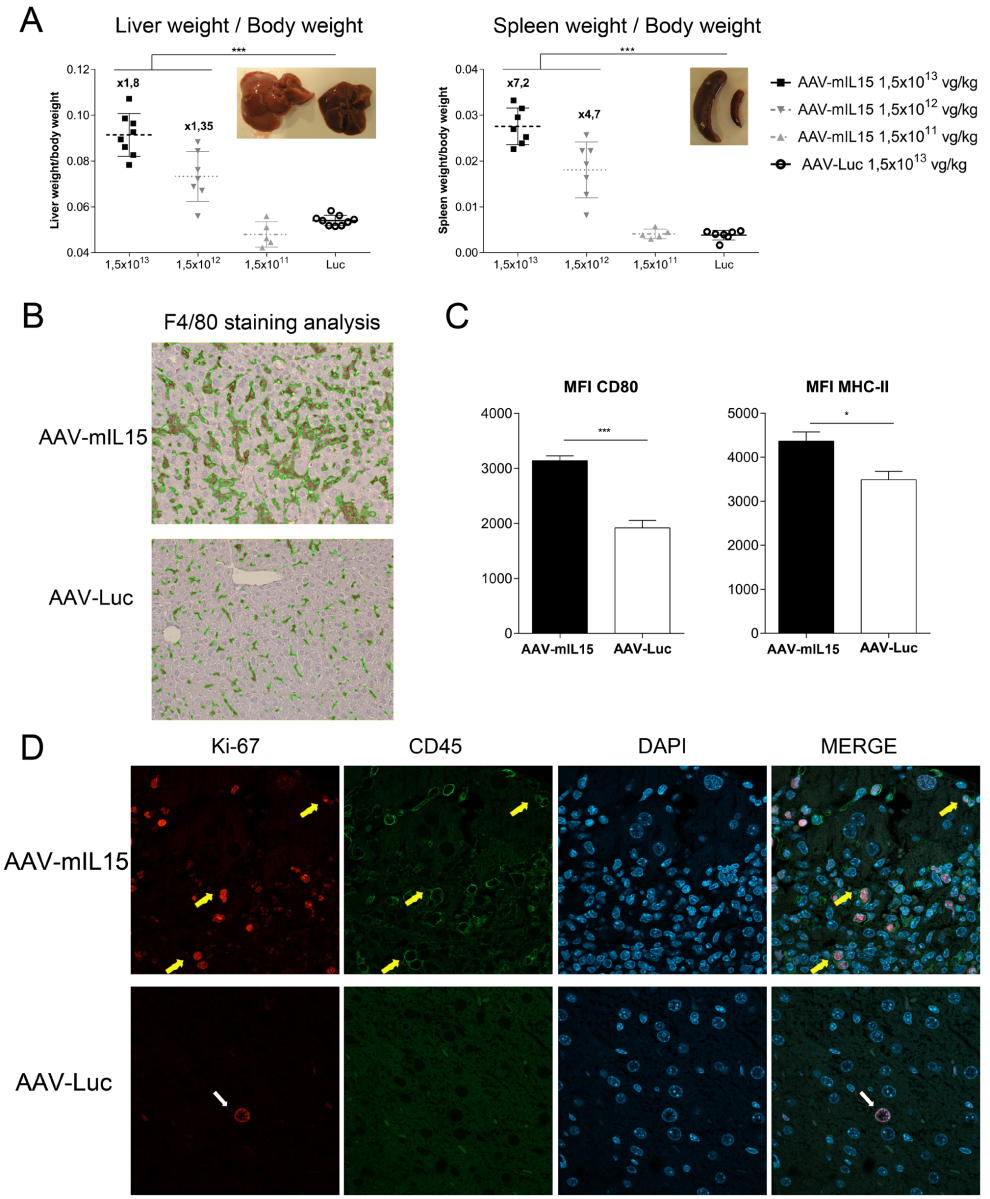
AAV-mIL15 administration is associated with the development of hepatosplenomegaly **A.** Twenty-one days after vector administration mice were sacrificed and the liver and spleen were weighed. The ratio between liver and spleen weight and body weight has been represented. Results are expressed as the mean ± SD. 6-8 mice per group. **B.** Livers were harvested immunohistochemical staining for F4/80 was performed. Representative images of the liver of one of the animals receiving the highest dose of each treatment were shown. **C.** Intrahepatic leucocytes were purified and CD80 an MHC-II expression was analyzed by flow cytometry on F4/80+ cells. Results are expressed as the mean ± SD of 6-8 animals per group. **D.** Immunofluorescence staining for Ki-67 and CD45 was performed; cell nucleus was stained using DAPI. Representative images of the liver of one of the animals receiving the highest dose of each treatment were shown.

When the C57BL/6 animals of the tumor experiment were sacrificed we observed that the group treated with AAV-mIL15 developed hepatomegaly and splenomegaly. To further investigate the effects of long-term exposure to mIL-15 we performed a more extensive characterization. C57BL/6 male mice (*n* = 8) were injected intravenously with three different doses of AAV-mIL15 1.5 × 10^11^, 1.5 × 10^12^, and 1.5 × 10^13^ vg/kg or 1.5 × 10^13^ vg/kg of AAV-Luc. Twenty-one days after AAV injection mice were sacrificed. Macroscopically we observed a significant increase in the size of the liver and spleen in the animals having received AAV-mIL15 compared to AAV-Luc-treated mice. In Figure 5A representative images of liver and spleen of a mouse having received the highest dose of AAV-mIL15 are shown in comparison to the same organs obtained from an AAV-Luc control animal. Both liver and spleen were weighed in all the animals, and, as shown in Figure 5A there is a significant and dose-dependent increase in the size of both organs in the AAV-mIL15-treated group. The analysis of liver histology shows the presence of an abundant leukocyte infiltrate in the liver of IL-15-treated mice that was dose-dependent (Supplementary Figure S2A). Furthermore, immunohistochemistry analysis of liver macrophages by anti-F4/80 staining indicated that IL-15 expression induced macrophage expansion and clusters formation (Figure 5B and Supplementary Figure S2B), suggesting macrophage activation that was confirmed by CD80 and MHC class I up-regulation (Figure 5C and Supplementary Figure S2C). In a previous paper IL-15 was described as a proliferation factor for hepatocytes [30], that might be one of the reason for hepatomegaly, however, Ki-67 expression analysis showed that IL-15 induced leucocyte- (CD45 positive cells) but no hepatocyte proliferation (Figure 5D). Serum biochemical analysis at day 21 revealed an increase in liver transaminases in the group of animals having received the highest doses of the AAV-mIL15 vector indicating a moderate liver damage (Supplementary Figure S2D).

### Long-term mIL-15 expression induces hematological stress and consequently the expansion of bone marrow hematopoietic precursors

**Figure 6:**
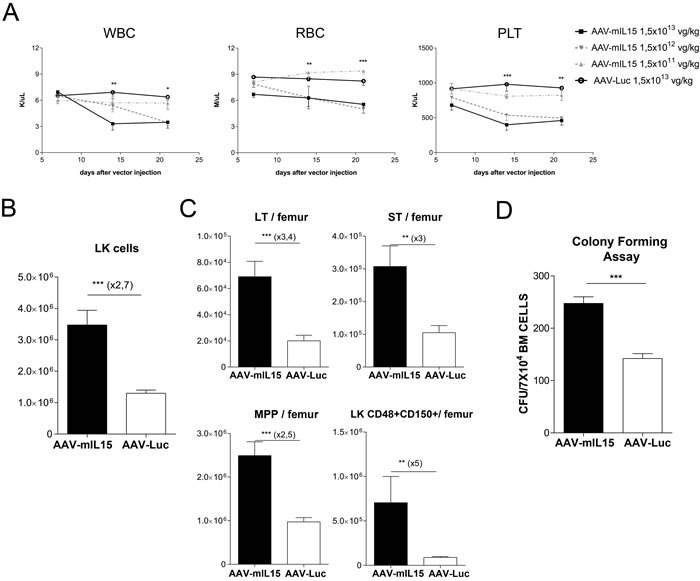
Analysis of hematological toxicity induced by long-term expression of mIL-15 **A.** Blood cell counts analysis was performed at the indicated time points after the administration of different doses of AAV-mIL15 vector. Results are expressed as the mean ± SD. **B.** Twenty-one days after vector administration, mice treated with a dose of AAV-mIL15 1.5×10^13^ vg/kg were sacrificed and bone marrow cells were examined for surface expression of Lin- and c-Kit to determine the number of LK cells per femur. Absolute number of LK cells in BM per femur was determined. Results are expressed as the mean ± SD. **C.** The numbers in the graph show immature (LT: LK, CD48-, CD150+) and more mature hematopoietic progenitor cells (ST: LK, CD48-, CD150-) and multipotent progenitors (MPPs: LK, CD48+, CD150-), or restricted progenitor cells (LK, CD48+, CD150+). Results are expressed as the mean ± SD of 4 mice per group. **D.** Number of colony forming units in the bone marrow of mice 21 day after treatment with AAV-mIL15 or AAV-Luc. Results are expressed as the mean ± SD of 6 mice per group.

To further characterize the side effects associated with high and sustained mIL-15 hepatic expression, we analyzed its effect on the hematopoietic system. Blood cell counts were evaluated weekly in animals treated as previously described. We found that sustained mIL-15 expression induced a reduction of leucocytes, platelets and red blood cells (Figure 6A). Homeostasis of blood cells is dependent on bone marrow hematopoietic stem cells (HSCs) possessing long-term self-renewal capacity. To determine the impact of IL-15 treatment on hematopoietic precursors, 21 days after treatment animals having received the highest dose of AAV-mIL15 were sacrificed and bone marrow (BM) was isolated from tibia and femur of each mouse and the number of HSCs was determined by flow cytometry. HSCs are cells expressing the stem cell markers c-kit and Sca-1 while lacking the canonical marker of lineage differentiation Lin (so-called LSK cells). The LSK cell population significantly increased in mIL-15-treated mice in comparison to Luc-treated mice (Supplementary Figure S3). However as we and others have previously demonstrated [31, 32], Sca-1 is an interferon-responsive molecule that is aberrantly up-regulated by IFN-α or IFN-γ on all hematopoietic progenitors preventing the use of this marker for HSC characterization. Using Lin- c-kit+ (LK) as identification markers for hematopoietic progenitor cells, our analysis revealed an increase of LK population in mice treated with AAV-mIL15 compared to AAV-Luc treatment (Figure 6B). The analysis of LK cells with anti-CD48 and anti-CD150, which discriminates between immature (LT: LK, CD48-, CD150+) and more mature progenitor cells (ST: LK, CD48-, CD150-) and multipotent progenitors (MPPs: LK, CD48+, CD150-), or restricted progenitor cells (LK, CD48+, CD150+) revealed a significant increase of all three populations in animals treated with AAV-mIL15 (Figure 6C). To determine the functionality of these hematopoietic progenitor cells we performed a colony forming unit (CFU) assay using bone marrow cells from AAV-mIL15 and AAV-Luc treated mice. As shown in Figure 6D, CFU numbers are significantly higher in AAV-mIL15 treated mice.

### IFN-γ is the main mediator of mIL-15-induced side effects

**Figure 7:**
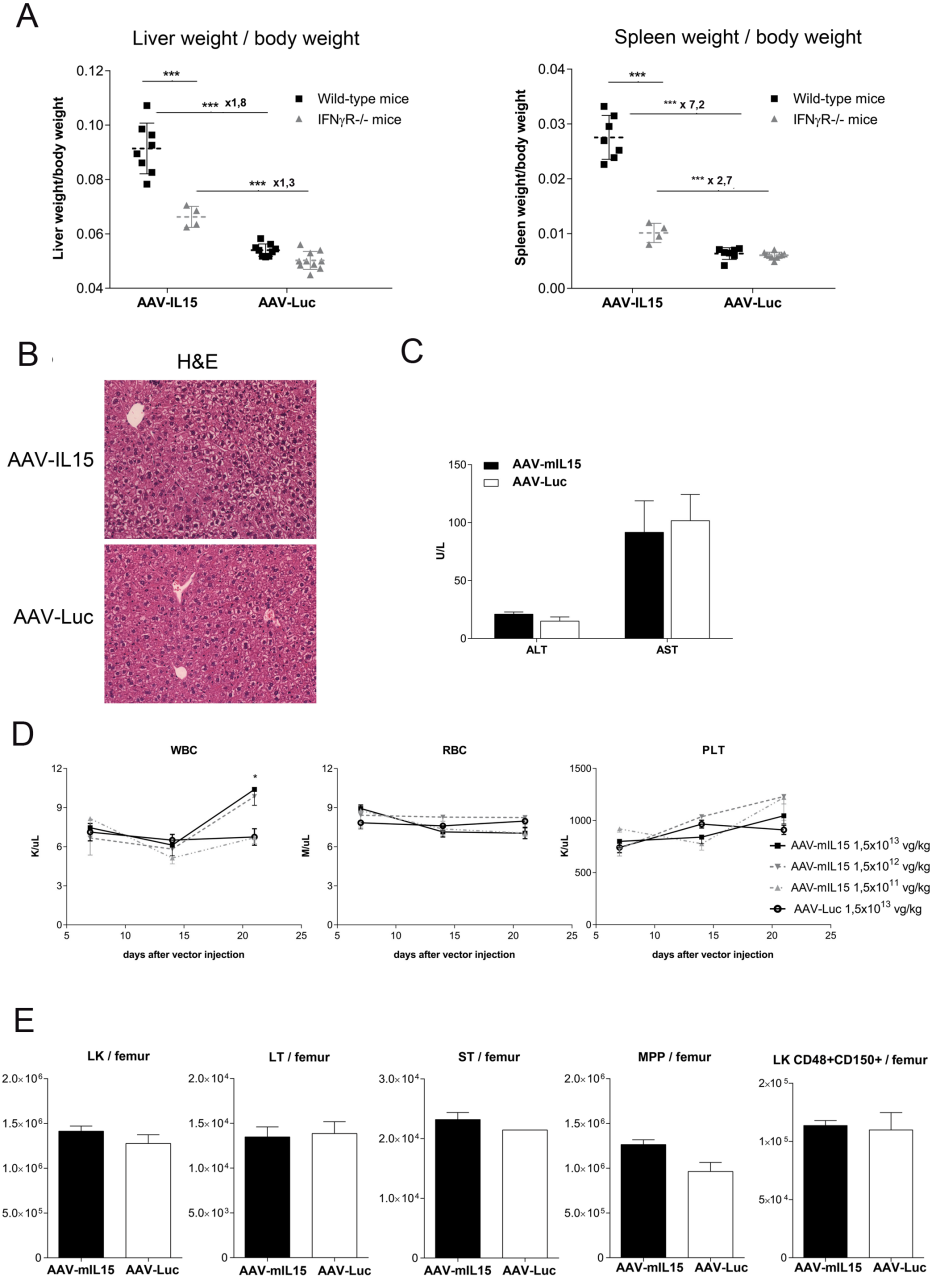
Analysis of liver and spleen weight and hematological parameters in IFNγR KO mice after AAV-mIL15 injection IFNγR−/− or C57BL/6 WT mice received 1.5 × 10^13^ of AAV-mIL15 or AAV-Luc (*n* = 4-8 mice per group). Twenty-one days after vector administration mice were sacrificed and **A.** the liver and spleen were weighed and compared. **B.** Livers were harvested and H&E was performed. Representative images of the liver of the animals receiving the highest dose of each treatment were shown. **C.** Serum biochemical analysis of ALT and AST. Results are expressed as the mean ± SD. **D.** Hematological analysis in IFNγR−/− mice treated with different doses of AAV-mIL15 or AAV-Luc. Results are expressed as the mean ± SD. 4-8 mice per group. **E.** Twenty-one days after vector administration mice were sacrificed and bone marrow cells were examined. Absolute number of LK cells in BM per femur was determined. Results are expressed as the mean ± SD the data obtained from 5 mice. LK cells were examined for surface expression of CD150 and CD48. The numbers in the graph show immature (LT: LK, CD48-, CD150+) and more mature progenitor cells (ST: LK, CD48-, CD150-) and multipotent progenitors (MPPs: LK, CD48+, CD150-), or restricted progenitor cells (LK, CD48+, CD150+). Results are expressed as the mean ± SD of 4 mice per group.

IL-15 acts on CD8- and NK cells inducing IFN-γ production [33, 34]. To analyze the role of this cytokine in the side effects associated to IL-15 exposure, mice deficient for the IFN-γ receptor (IFNγR−/−) were treated with 1.5 × 10^13^, 1.5 × 10^12^ or 1.5 × 10^11^ vg/kg of AAV-mIL15 or 1.5 × 10^13^ AAV-Luc. Twenty one days after injection animals were sacrificed and liver and spleen were weighed (Figure 7A and Supplementary Figure S4). Although the size of both organs significantly increased in IFNγR−/− after IL-15 treatment, the fold increase was significantly lower in IFNγR−/− than in wild-type mice (Figure 7A). At the highest dose of AAV-mIL15 the size of the liver increases 80% in wild-type mice but only 30% in IFNγR−/− mice and the spleen size increases 620% in WT mice and only 170% in IFNγR−/− mice pointing towards IFN-γ as the main mediator of IL-15-induced hepatomegaly and splenomegaly, although other factors induced by IL-15 seems to be involved. The histological analysis of the liver revealed no increase in inflammatory infiltrate in the liver of IFNγR−/− receiving mIL-15 (Figure 7B). Moreover, serum biochemical analysis at day 21 revealed normal levels of liver transaminases indicating the absence of damage in IFNγR−/− mice (Figure 7C). Furthermore, blood cell count analysis revealed no reduction of leucocytes, platelets and red blood cells in IFNγR−/− indicating that mIL-15 hematological toxicity is IFN-γ-dependent (Figure 7D). Interestingly an increase on leucocyte number was observed (Figure 7D left). The analysis of hematopoietic progenitor cell populations in the BM of IFNγR−/− mice treated with AAV-mIL15 reveals that in the absence of IFN-γ receptor there is no expansion of these populations (Figure 7E).

### mIL-15 mediated side effects are mainly mediated by IFN-γ producing T cells

**Figure 8:**
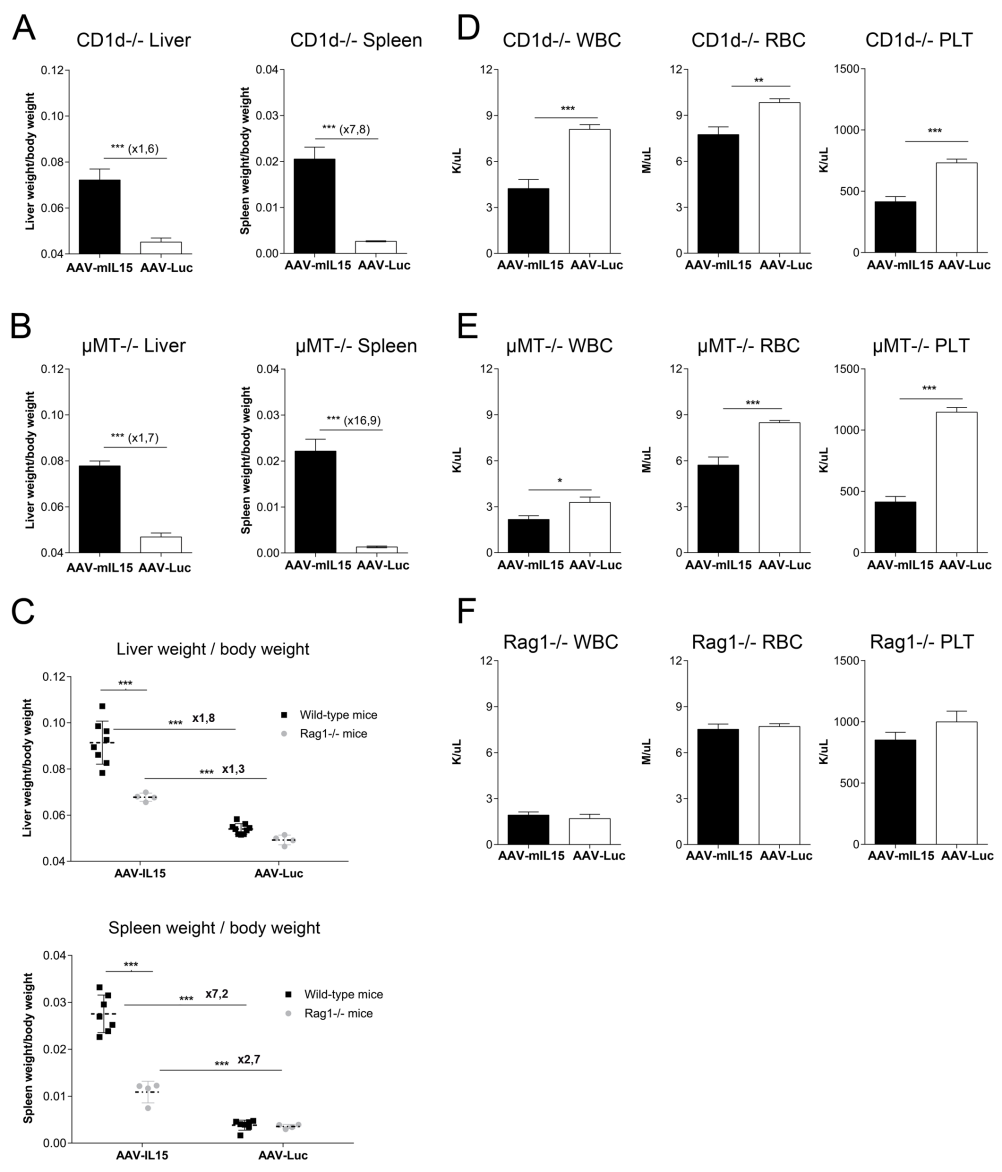
Analysis of splenomegaly, hepatomegaly and hematological toxicity in CD1d-, μMT- and RAG1- deficient mice after AAV-mIL15 expression. CD1d −/−, μMT−/− and RAG1−/− male mice received 1.5 × 10^13^ of AAV-mIL15 or AAV-Luc. **A.**-**C.** Twenty-one days after vector administration mice were sacrificed and the liver and spleen were weighed A. CD1d−/−, B. μMT−/− and C. Comparative analysis of liver (up) and spleen (down) weight in wild-type and RAG1−/− mice 21 days after treatment with 1.5 × 10^13^ vg/Kg of AAV-mIL15 or AAV-Luc. Results are expressed as the mean ± SD of 6-8 mice per group. Hemogram analysis was performed in **D.** CD1d−/−, **E**. μMT−/− and **F**. RAG1−/− mice. Results are expressed as the mean ± SD of 6-8 mice per group.

To analyze the cells involved in mIL15-mediated side effects RAG1−/−, CD1d−/−, and μMT−/−, were injected with AAV-Luc or AAV-mIL15 at a dose of 1.5 × 10^13^ vg/kg, and 21 days after treatment liver and spleen size was analyzed and cell blood counted. As shown in Figure 8A and 8B, in CD1d−/− and mMT−/− the liver and spleen weight increased like in wild-type mice after AAV-mIL15 treatment, thus demonstrating that neither NKT- nor B-cells are responsible for the increase in organ size. However, RAG1−/− treated with AAV-mIL15 presented with a less pronounced hepatosplenomegaly in comparison to wild-type mice (Figure 8C), indicating that IFN-γ-producing T cells but not NK cells are the main cell type responsible for liver and spleen enlargement.

Furthermore, analysis of blood cell counts showed that while CD1d and μMT-deficient mice had a significant reduction of blood cell populations as observed in wild-type mice (Figure 8D and 8E), no reduction in blood cell counts were observed in RAG1−/− mice, indicating together with the results obtained in IFNγR−/− that the hematological stress caused by mIL-15 is mediated by the action of IFN-γ producing T cells (Figure 8F). Analysis of HSC in the BM of CD1d−/− and μMT−/− mice revealed an increase in the LK population in mice treated with AAV-mIL15 as shown in WT mice (Supplementary Figure S5A and B). Instead RAG1−/− mice showed that in the absence of T cells there is no expansion of LK population (Supplementary Figure S5C). These data further indicate that the expansion of hematopoietic progenitors is more likely due to the hematological stress induced by mIL-15 rather than to a direct effect of mIL-15 on hematopoietic precursor cells.

**Figure 9:**
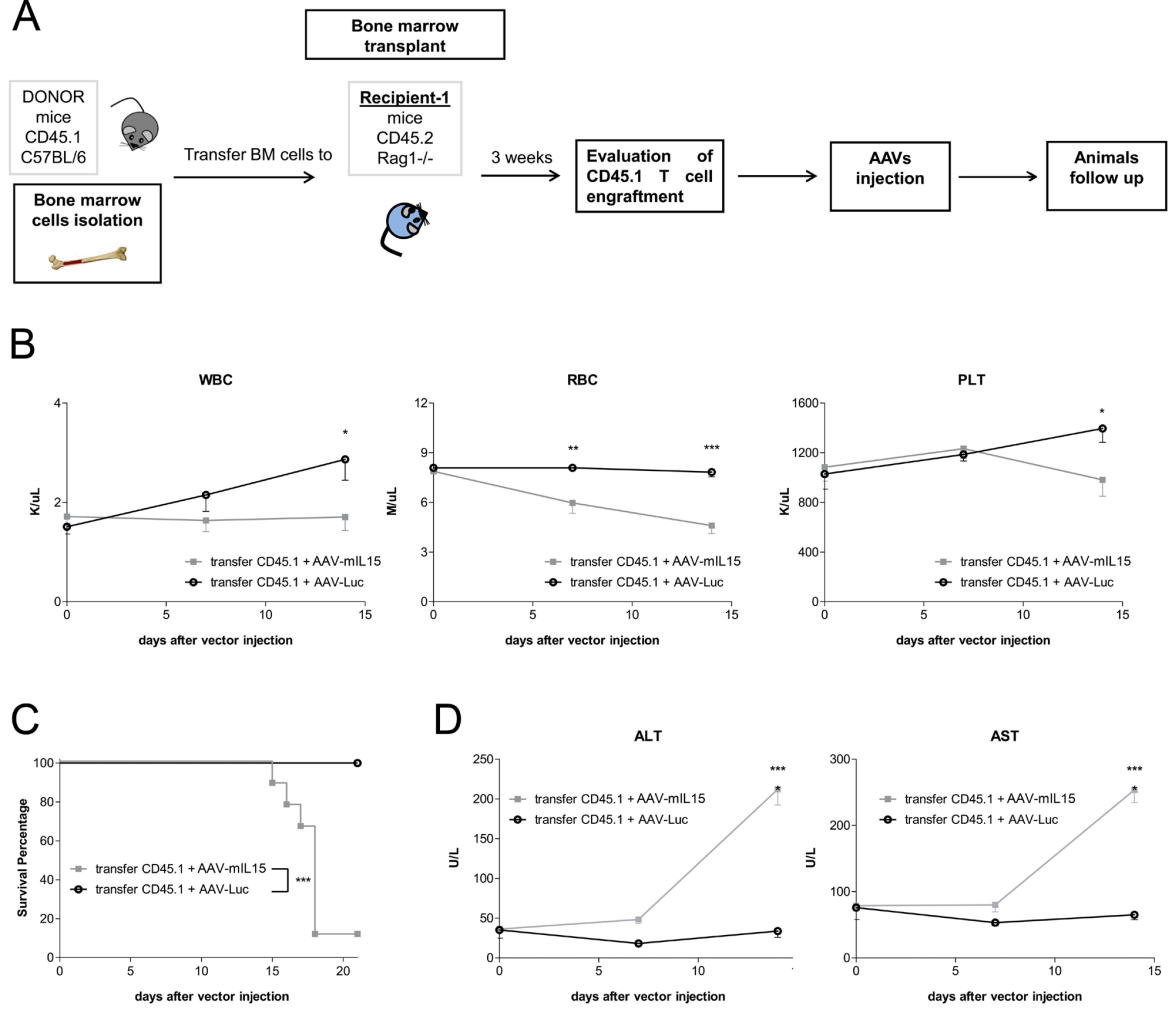
Bone marrow cell transfer from WT mice to RAG1−/− mice restores mIL-15 mediated side effects **A.** Schematic representation of the experimental design. **B.** Blood cell counts analysis was performed at the indicated time points after the administration of AAV-mIL15 or AAV-Luc vector. Results are expressed as the mean ± SD of 8 mice. **C.** Kaplan-Meier survival analysis of RAG1−/− mice receiving bone marrow cells from C57BL/6 mice and AAV-mIL15 or AAV-Luc. (***p <0.001). **D.** Serum biochemical analysis of ALT and AST. Results are expressed as the mean ± SD, *n* = 8.

To further confirm the role of T cells in IL-15 mediated side effects we transferred bone marrow cells from C57BL/6 CD45.1+ mice to RAG1−/− CD45.2+ mice. After confirming CD45.1 T cells engraftment in RAG1−/− mice, mice were treated with AAV-mIL15 or AAV-Luc (Figure 9A). Blood cell counts were evaluated weekly starting at day 7. We found that sustained IL-15 expression induced a reduction of leucocytes, platelets and red blood cells in RAG1−/− mice that had received WT bone marrow and AAV-mIL15 but no AAV-Luc (Figure 9B). Interestingly, at day 21 only 1 of the RAG1−/− that have received BM cells from WT mice and AAV-mIL15 remained alive indicating a strong toxic effect of mIL-15 administration in these mice (Figure 9C). Furthermore, we observed hepatomegaly and splenomegaly in the animals receiving mIL15 (data not shown) and as observed in WT mice mIL-15 induced liver injury in animals receiving BM transplant as evidenced by the elevation of liver transaminases (Figure 9D).

## DISCUSSION

IL-15 has been proposed as an alternative to the use of IL-2 in cancer therapy. Although both cytokines share many biological properties, IL-15 shows some interesting functional particularities, such its capacity to activate T-cells [8], to inhibit activated induced cell death (AICD) of T-cells [9] and protect T effector cells from the action of T regulatory cells [35]. These properties suggest a better capacity of IL-15 to induce antitumoral immune responses than IL-2. Furthermore, IL-15 has been demonstrated to be less toxic than IL-2 in different preclinical animal models, demonstrating a better efficacy *versus* toxicity profile [36]. For these reasons several clinical trials are ongoing testing its antitumoral activity [22]. However, there is limited information regarding the antitumoral efficacy and the safety of the native version of mIL-15 in mice, since most of the studies have been performed using human IL-15 (hIL-15) or a modified version of the cytokine in which the natural IL-15 signal peptide has been replaced by a more efficient signal peptide enhancing its secretion. Although hIL-15 is active on murine cells, the aminoacid identity between hIL-15 and just mIL15 is of 73%. In fact, while the binding affinity of hIL-15 and mIL-15 to murine IL-15Rα subunit is very similar they significantly differ in the binding affinity to the IL-2Rβγ_c_ subunits, and mIL-15 was reported to be 200-fold more active than hIL-15 *in vitro* [37]. These data suggest that the studies performed using hIL-15 in mice are far to be predictive of the activity and toxicity of this cytokine in humans. The small number of studies performed to test the antitumoral activity of the recombinant murine IL-15 protein showed only marginal tumor effects [17, 38]. This could be due to several reasons, one of them being the improper presentation of the cytokine (unbound to the IL-15Ra), or the short half-life of IL-15 in serum. To solve both issues we have developed an AAV vector expressing murine IL-15, carrying its native signal peptide, from the hepatocytes in which IL-15Rα subunit is expressed.

Here we present that mice treated with AAV-mIL15 showed sustained and vector dose-dependent levels of the IL-15/IL-15Rα complex in serum, which is associated with the production of IFN-γ. Moreover, long-term mIL-15 expression determines a strong activation and expansion of CD8 T cells in both, the liver and in the spleen. Thus, our data indicate that AAV-mediated long-term mIL-15 expression induces a potent activation of the immune system. Interestingly, we observed a reduction of NK cells from all the organs/tissues analyzed that we believe is associated to the sustained presence of mIL-15, more experiments should be performed to clarify this unexpected observation. Interestingly, transgenic mice overexpressing IFN-γ shows impaired NK cell development and a profound reduction of NK cells in spleen supporting our data and suggesting a role for IL15-induced IFN-γ production in the disappearance of NK cells [39].

When we tested the antitumoral efficacy of AAV-mIL15 in a murine model for colorectal metastasis (in which CD8 T cells play a major role in tumor growth inhibition), we found a very moderate antitumoral effect with a small reduction in tumor size despite a significant increase in survival. Our results are consistent with previous studies in mice showing a moderate antitumoral activity of mIL-15 when is administered alone [40], that can be improved if combined with other cytokines [40, 41]. Moreover, IL-15 is known to activate negative regulatory checkpoints so the combination with antibodies to block cytotoxic T lymphocyte antigen 4 (CTLA-4) and programmed death ligand 1 (PD-L1) resulted in an improvement of the antitumoral activity that should be carefully evaluated [42].

However, what was very surprising to us was the observation made regarding important secondary effects developed in AAV-mIL15-treated mice, not previously reported, except for a particular study in which hydrodynamic injection of a plasmid expressing IL-15 bound to the sushi domain and stabilized by APOA1 molecules induces lethal acute lymphocytic pneumonitis [43]. In the present study all the animals developed splenomegaly and hepatomegaly in a vector dose-dependent manner. The abnormal growth of both organs was associated with massive infiltration of proliferating lymphocytes and macrophages, cell hyperplasia and a moderate liver damage. Interestingly, hepatosplenomegaly was previously observed in rhesus macaques treated with rhIL-15 for 12 days, where a significant liver leukocyte infiltrate was observed [44].

Additionally we found that AAV-mIL15 treatment was associated with the induction of significant hematological stress. Mice developed anemia, thrombocytopenia and leukopenia inducing the activation of hematopoietic precursor proliferation in the bone marrow in an effort to recover blood cell counts. Bone marrow hypocellularity, anemia and severe neutropenia have been observed in macaques treated daily with human recombinant IL-15 by subcutaneous injection [45] and, more importantly, recently similar side effects have been observed in first in human clinical trial of recombinant-IL15. All the patients treated with the highest dose of rhIL15 developed liver damage and lymphopenia; in some cases associated with thrombocytopenia, anemia or neutropenia, indicating that our data somehow mimic the effect of IL-15 in patients, probably being more dramatic due to the sustained exposure to the cytokine.

Experiments performed in IFN- receptor- and in RAG1-deficient mice suggest that the main mediators of IL-15-induced side effects are IFN–producing T cells. Taking together T-cell-derived IFN-γ play a prominent role in IL-15 mediated toxicityRegarding splenomegaly and hepatomegaly other cytokines induced by IL-15 like TNF-α or IL-18 might also be implicated [46]. Moreover, first-in-man phase I clinical trial also showed that IL-6 is markedly induced following IL-15 injection. This cytokine is a major promoter of inflammation and can cause severe toxicity. We cannot discard a role of this cytokine in the pathology developed by AAV-mIL15 treated mice [22].

However, the hematological stress associated to IL-15 was essentially mediated by IFN–producing T cells. Furthermore, although it has been suggested that IL-15 might have a direct effect on bone marrow hematopoietic stem cell [47], our data indicate that IL-15 itself fails to induce more immature progenitor expansion and that this effect is mediated by the hematological stress induced by IFN-γ producing T cells. It has been recently shown that IFN-γ plays a major role in LSK expansion [39, 48, 49], however, in our experimental system the fact that no expansion was detected in T cell-deficient mice indicates that this expansion is mainly due to the haematological stress.

IL-15 is a cytokine subjected to an extraordinarily strict control; its expression is controlled at different levels: transcription, translation, and intracellular trafficking [50]. The toxicity associated with long-term exposure to IL-15 seen in our study highlight the necessity for this tight control. Properties of IL-15, such as its capacity to activate CD8 and NK cells or to inhibits AICD, initially seen as beneficial for the use of this cytokine as an antitumoral agent, can be detrimental when there are no mechanism of down-regulation.

In summary, although IL-15 is not associated with the undesirable features of IL-2 [3, 4], our data demonstrated that sustained expression of high levels of this cytokine induce important side effects involving the hematopoietic system and also important organs like liver and spleen. As shown in this work, all these effects are mainly mediated by T cells producing IFN-γ. Based on these results we can conclude that for future clinical applications of IL-15 the dose and the duration of the cytokine should be carefully controlled and in the case of the development of undesirable side effects IFN–blocking agents might be useful. However, the use of IFN–blocking agents could block or decrease the effector functions of the CD8+ T and NK cells which are the major anti-tumor effectors populations, for this reason the use of inducible promoters might represent a more attractive strategy for IL-15 immuno-gene-therapy applications [51] to maintain the antitumoral activity and reducing the negative effects of overexpression of this cytokine.

We believe our findings have important implications for the design of future clinical trials using IL-15 as an antitumoral agent.

## MATERIALS AND METHODS

### Animals and treatment

Female 8-weeks-old C57BL/6 mice were purchased from Harlan Laboratories (Barcelona, Spain). IFN-γ receptor deficient mice (IFNγR−/−), B, T and NKT deficient mice (RAG1−/−), NKT deficient mice (CD1d−/−) and B cell deficient mice (μMT−/−) all of them in C57BL/6 background, were bred and maintained under pathogen-free conditions in the animal facility of the University of Navarra. All procedures were approved by the Ethical Committee for Animal Testing of the University of Navarra.

Mice were injected intravenously with the AAV viruses. Animals were anesthetized by intraperitoneal injection of a mixture of xylacine (Rompun 2%, Bayer) and ketamin (Imalgene 500, Merial) 1:9 v/v. Blood collection was performed by bleeding from the retro-orbital plexus, and serum samples were obtained by centrifugation of total blood.

### Viral construction, production and purification

The expression cassette contained in the AAV-mIL15 vector consists of the murine interleukin-15 gene (GenBank accession number DQ083237.1) carrying the long (48 aa) signal peptide, under the regulation of a chimeric liver specific promoter composed of the human α1-antitrypsin (AAT) promoter with regulatory sequences from the albumin enhancer (Ealb) [27]. Control virus expressed the reporter gene luciferase, AAV-Luc. rAAV8 vectors were produced as previously described [51]. Viral titers in terms of viral genome per milliliter (vg/ml) were determined by quantitative-PCR (Q-PCR).

### Determination of murine IL-15 and IFN-γ

Concentration of murine IL-15 was determined by Mouse IL-15R/IL-15 Complex Elisa Ready-SET-GO! (eBiosciences) and concentration of murine IFN-γ was determined by Mouse IFN-γ ELISA Kits (BD Bioscience-Pharmigen, San Diego, CA) according to manufacturer's instructions.

### Histology and immunohistochemistry analysis

Liver sections were fixed in 4% paraformaldehyde (Panreac), embedded in paraffin, sectioned (5 μm), and stained with hematoxylin and eosin. Sections were mounted and observed by light microscopy. Immunohistochemical staining for Ki-67 (rabbit monoclonal, clone SP6, 1:100; NeoMarkers, RM-9106) and F4/80 (rat anti-mouse, 1:400; eBiosciences, 14-4801-82) was performed using the EnVision^TM^+ System (Dako, Glostrup, Denmark) according to the manufacture's recommendations. Sections were revealed with 3,3′-diaminobenzidine (DAKO, Barcelona, Spain), counterstained with Harris' hematoxylin and mounted with DPX (Merck SL, Madrid, Spain) stained. Image acquisition was performed on the Zeiss Axio Imager M1 microscope with the AxioCam ERC 5s camera using the ZEN Pro software, all from Carl Zeiss Inc. (Thornwood, NY).

### Tumor models

For subcutaneous tumor formation, a total of 10^6^cells were injected in the right hind flank. Hepatic tumor were established by direct implantation of 5×10^5^ MC38 cells in the left liver lobe in isofluorane-anaesthetized animals C57BL/6 mice following medial laparotomy. Cells were resuspended in a total volume of 50 μl saline solution.

Tumor progression was monitored by direct measurement using caliper for subcutaneous tumor and using echography obtained images for intrahepatic tumor. Volumes were calculated using the formula V = 0.5 × D × d^2^, where D and d are the maximum and minimum tumor diameters respectively. Survival was checked daily and when necessary, mice were euthanized using a mixture of O_2_ and CO_2_.

### Hemogram

Blood cell counts were analyzed using an automated veterinary haematological analyser with a pre-programmed murine calibration mode (Hemavet 950FS; Drew Scientific, Waterbury, CT).

### ALT and AST analysis

Serum alanine aminotransferase (ALT) and aspartate aminotransferase (AST) activities were analyzed in a Hitachi Automatic Analyzer (Boehringer, Indianapolis, IN).

### Cells isolation

For bone marrow cell isolation bones were flushed using a 23-gauge (femurs and tibias) or 26-gauge needle (iliac crests) and the bones discarded. The cells were obtained by mechanical disruption and washed by centrifuging at 1300rpm for 5 minutes at 4°C, resuspended in PBS, and then filtered through a 70-μm filter. Red blood cells were removed using a lysis buffer. Cell concentrations were determined with an automatic cell counter (Z1 Coulter Particle Counter, Beckman Coulter). Splenocytes were washed in cell culture medium (RPMI 1640) and filtered through a 70μm nylon cell strainer. Cell concentrations were determined with an automatic cell counter and splenocytes were adjusted to the desired final concentration.

For the isolation of intrahepatic mononuclear cells the abdomen of mice was opened and portal vein was cannulated with a 24G intravenous catheters and perfused with 10 ml of Ca^2+^- and Mg^2+^-free phosphate buffer solution preheated to 37°C. After the perfusion the cava vein was cut and liver extracted. The liver was incubated for twenty minutes in 10 mL of PBS solution containing 1000 units of type II Collagenase (Gibco). The liver was passed through a 70 μm nylon cell strainer. The cell suspension was centrifuged at 1200 rpmfor 10 min and the cell pellet was resuspended in 40% Percoll and centrifuged at 1800 rpmfor 10 min. RBC were removed using a RBC lysis Buffer. Cell pellet was resuspended in RPMI 1640 medium to the desired final concentration.

### CD8, CD4, NK staining

Single-cell suspensions were pretreated with FcR-Block (anti-CD16/32 clone 2.4G2; BD Bioscience-Pharmigen). Afterward, cells were stained for: CD8a (Pacific Blue-conjugated antimouse CD8. clone 53.6.7, Biolegend), CD4 (APC/Cy7-conjugated antimouse CD4 cloneGK1.5; Biolegend), NK-1.1 (APC-conjugated antimouse NK1.1, clone PK136, Biolegend), CD69 (FITC conjugated antimouse anti-CD69 clone H1.2F3; eBioscience).

### Hematopoietic stem cell (HSC) staining

Whole bone marrow cells were isolated and stained on ice with various antibody cocktails to identify each progenitor compartment. Hematopitic stem cells (HSC) also name LSK cells were stained for Lin (FITC or PE-antimouse lineage antibody cocktail, Biolegend) (APC-antimouse lineage antibody cocktail, BD Biosciences), c-Kit^+^(PE- or APC/Cy7-antimouse CD117, Biolegend) and Sca-1 (Pacific Blue-antimouse Sca-1. Biolegend). To identify more primitive cells Long Term (LT: LK, CD48-, CD150+), Short Term (ST: LK, CD48-, CD150-), Multipotent progenitor cells (MPPs: LK, CD48+, CD150-) and restricted progenitor cells (LK, CD48+, CD150+) LSK cells were additionally analysed for CD48 (FITC-antimouse CD48 from Biolegend) and CD150 (PE/Cy7-antimouse CD150 from Biolegend) expression.

### FACS

For flow cytometry analysis the 8-color BD FACS Canto II or the 4-color FACSCalibur were used. Cells were analyzed with FlowJo and DIVA software.

### Automated analysis of F4/80 immunohistochemistry

To analyse F4/80 expression digital images were acquired with an AxioImager. MI microscope (Zeiss, Germany) using an in-house Metamorph macro (Molecular Devices, USA). All images were stored in uncompressed 24-bit colour TIFF format. Images were automatically analysed using a plugin developed for Fiji, ImageJ.41.

### CFU assay

CFU assays were performed according to the manufacturer's instructions (Stem Cell Technologies) using Mouse Methylcellulose Complete Media (HSC007). BM cells were plated at 7 × 10^4^ cells in 6-well plates and incubated at 37°C in a humidified atmosphere of 5% CO2. Cells were cultured for 15 days and monitored by phase-contrast microscopy.

### Bone marrow transplantation

BM cells from CD45.1+ C57BL/6 (5 × 10^6^ cells) were isolated and transplanted by intravenous injection into C45.2 RAG1−/− recipient mice.

### Statistical analysis

The data are presented as mean values ± standard deviation and were analyzed for significance by the Student *t* test (*p* < 0.05 was considered significant). Differences in parameters among groups were analyzed with one-way ANOVA followed by Bonferroni multiple-comparison test with the GraphPad Prism 6.0 software. Survival curves data analysis was made by a Kaplan-Meier test and statistical analysis was done with the log rank test.

## SUPPLEMENTARY MATERIALS FIGURES AND TABLES



## Abbreviations

AAV: Adeno-associated virus
IFN-γ: interferon-γ
IL-2: interleukin-2
IL-15: interleukin-15
IL-15Rα: IL-15 Receptor α subunit
TNF-α: tumor necrosis Factor-α
ALT: alanine aminotransferase
AST: aspartate aminotransferase
HSCs: hematopoietic stem cell
MPP: multipotent progenitor cells
