# Supplementary material for: Identification of IFN-γ-producing T cells as the main mediators of the side effects associated to mouse interleukin-15 sustained exposure
Source: Oncotarget. 2016 Jun 23;7(31):49008–26. doi: 10.18632/oncotarget.10264 (PMC5226487; doi:10.18632/oncotarget.10264)
Supplement: Supplementary file 1 [file oncotarget-07-49008-s001.pdf]

Identification of IFN-γ-producing T cells as the main mediators of the side effects associated to mouse interleukin-15 sustained exposure

Supplementary Material

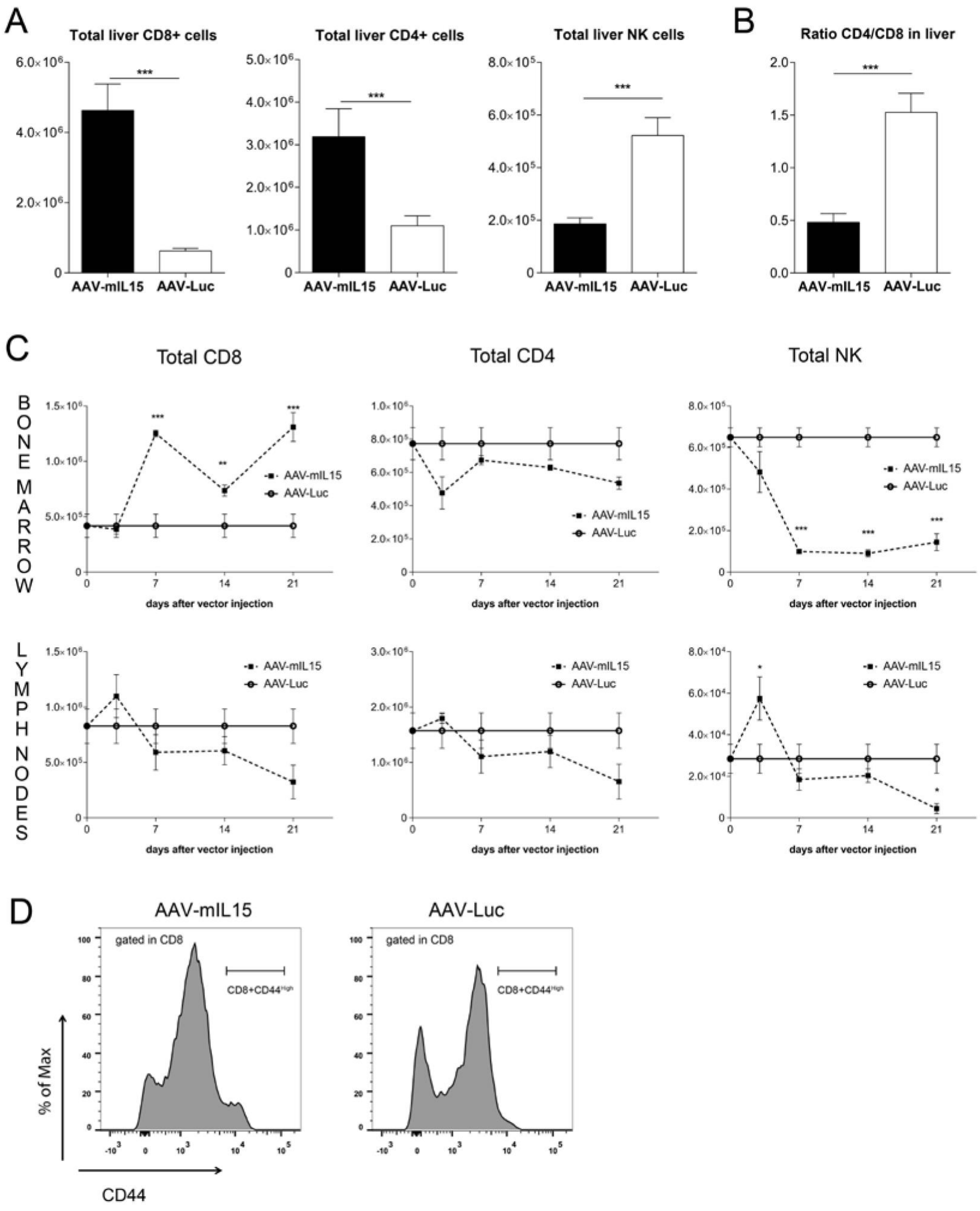

Supplementary Figure 1. **Analysis of lymphocyte subsets after administration of AAV-mIL15. A**. Twenty-one days after the administration AAV-mIL15 or AAV-Luc at a dose of 1.5 x 10^{13} vg/kg mice were sacrificed and the number of CD8, CD4 and NK cells obtained from the liver was analyzed by flow cytometry. **B**. The ratio CD4/CD8 is shown. Results are expressed as the mean ± SD of 6-8 mice per group. **C.** 3, 7, 14, and 21 days after the administration AAV-mIL15 or AAV-Luc at a dose of 1.5 x 10^{13} vg/kg mice were sacrificed and the number of CD8, CD4 and NK cells obtained from the bone marrow and lymph nodes was analyzed by flow cytometry. **D.** The activation status of CD8 was determined by analyzing the expression of CD44. Representative flow cytometry histograms of a mouse from each group are shown.

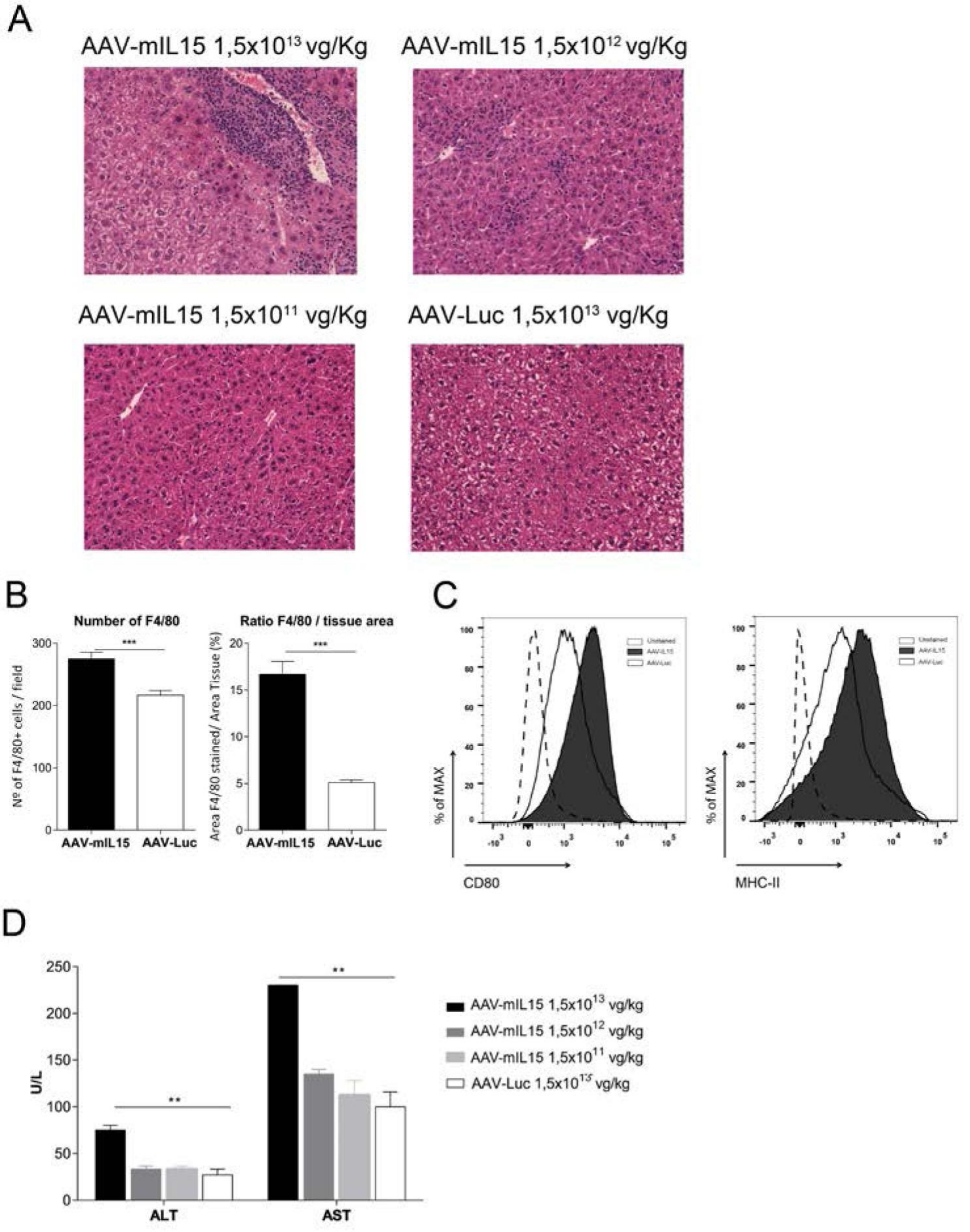

Supplementary Figure 2. **Characterization of intrahepatic leukocyte population and liver damage of mice receiving AAV-mIL15 or AAV-Luc. A**. C57BL/6 male mice (n=8) were intravenously injected with three different doses of AAV-

mIL15 1.5 x 10^{11}, 1.5 x 10^{12}, and 1.5 x 10^{13} vg/Kg or 1.5 x 10^{13} vg/Kg of AAV-Luc. Twenty-one days after AAV injection mice were sacrificed. Livers were harvested and H&E staining was performed. Representative images one of the animals per group were shown. **B**. Liver sections obtained from mice treated with 1.5 x 10^{13} vg/Kg of AAV-mIL15 or AAV-Luc for 21 days were analysed by F4/80 Immunohistochemestry using an anti-F4/80 antibody. The number of F4/80 positive cells and the area stained by the F4/80 antibody were quantified. Results are expressed as the mean ± SD of 8 mice per group. **C**. The activation status of F4/80 positive cells obtained from mice treated with 1.5 x 10^{13} vg/Kg of AAV-mIL15 or AAV-Luc for 21 days was determined by analyzing the expression of CD80 and MHC class II (MHCII). Representative flow cytometry histograms of a mouse from each group are shown. **D**. Serum biochemical analysis of ALT and AST. Results are expressed as the mean ± SD of 6-8 mice per group.

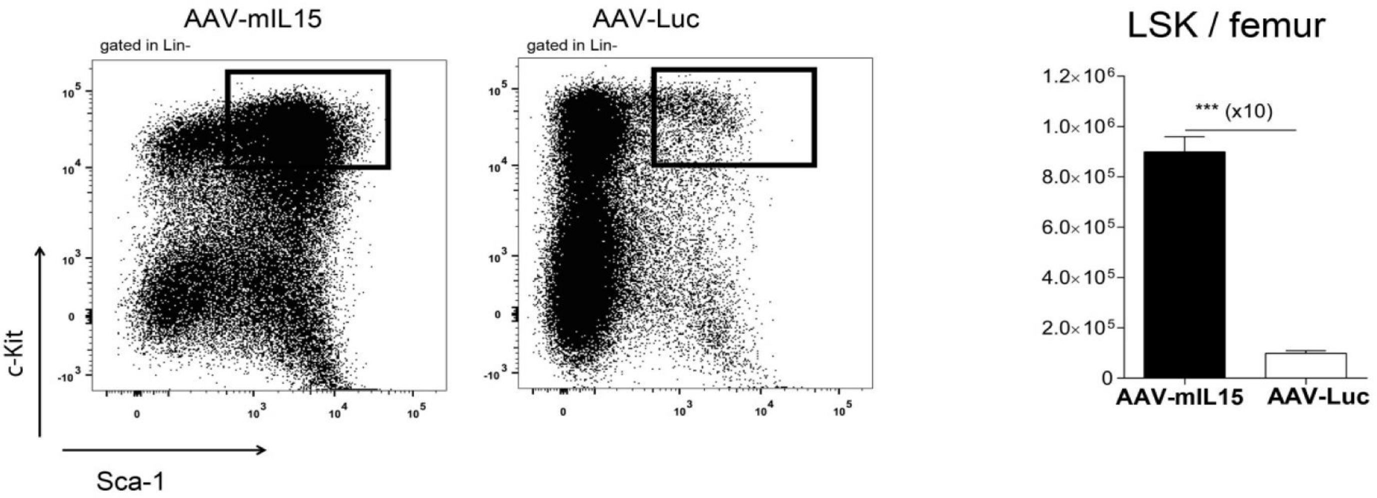

Supplementary Figure 3. Long term IL-15 expression induces expansion of LSK population in bone marrow. (left) Representative flow cytometry analysis of Lineage negative (Lin-) cKit+ and Sca-1+ cells (LSK) of BM cells obtained from mice treated with 1.5 x 10^{13} vg/Kg of AAV-mIL15 or AAV-Luc. The analysis was performed three weeks after vector injection. (right) Bar graphs represent the quantitative analysis of numbers of LSK (left). Results are expressed as the mean ± SD of 4 mice per group

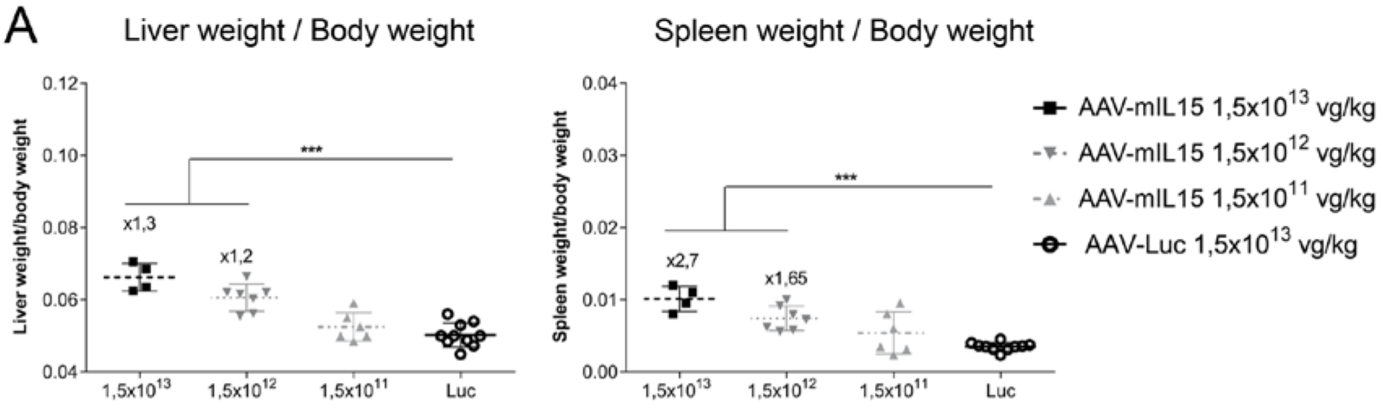

Supplementary Figure 4. Analysis of liver and spleen weight parameters in IFNγR KO mice after AAV-mIL15 injection. IFNγR-/- received 1.5×10^{13}, 1.5×10^{12} or 1.5×10^{11} ^{v} vg/kg of AAV-mIL15 or 1.5×10^{13} AAV-Luc (N=4-8 mice per group). Twenty-one days after vector administration mice were sacrificed and the liver and spleen were weighed and compared.

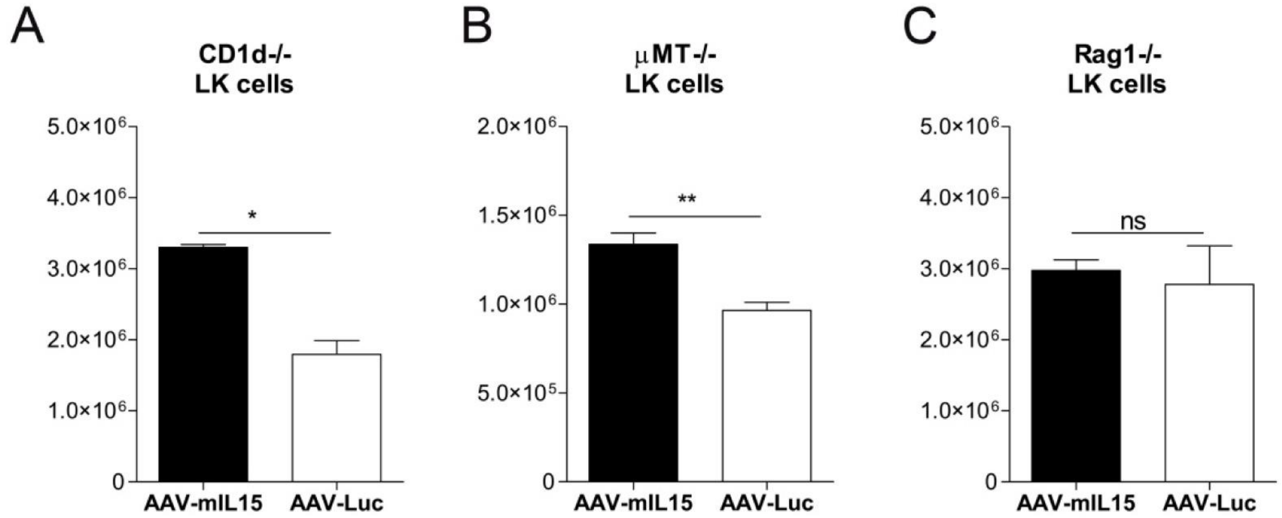

Supplementary Figure 5. Analysis of LK population in the bone marrow of different mice strain. Twenty-one days after vector administration, mice treated with a dose of AAV-mIL15 or AAV-Luc $1.5x10^{13}$ vg/kg were sacrificed and bone marrow cells were examined for surface expression of Lin- and c-Kit to determine the number of LK cells per femur. Absolute number of LK cells in BM per femur was determined in CD1d-/- **A**., μMT-/- **B**. and Rag1-/- **C**. mice. Results are expressed as the mean ± SD of 4-5 mice per group.
